# Facile Ag-Film Based Surface Enhanced Raman Spectroscopy Using DNA Molecular Switch for Ultra-Sensitive Mercury Ions Detection

**DOI:** 10.3390/nano8080596

**Published:** 2018-08-06

**Authors:** Xiujie Liu, Mengmeng Liu, Yudong Lu, Changji Wu, Yunchao Xu, Duo Lin, Dechan Lu, Ting Zhou, Shangyuan Feng

**Affiliations:** 1 Key Laboratory of Optoelectronic Science and Technology for Medicine of Ministry of Education, Fujian Provincial Key Laboratory of Photonics Technology, Fujian Normal University, Fuzhou 350007, China; m18396517281@163.com (X.L.); MM_liu@163.com (M.L.); m13512027287_1@163.com (Y.X.); linduo1986@163.com (D.L.); 2 Fujian Key Laboratory of Polymer Materials, College of Chemistry and Materials Science, Fuzhou 350007, China; 18060489009@163.com (C.W.); 18149544204@163.com (D.L.); zt1102801305@163.com (T.Z.)

**Keywords:** surface-enhanced Raman spectroscopy (SERS), Ag-film, Hg^2+^ ions detection, SERS sensor

## Abstract

Heavy metal pollution has long been the focus of attention because of its serious threat to human health and the environment. Surface enhanced Raman spectroscopy (SERS) has shown great potential for metal detection owing to many advantages, including, requiring fewer samples, its minimal damage to specimen, and its high sensitivity. In this work, we proposed a simple and distinctive method, based on SERS, using facile silver film (Ag-film) combined with a DNA molecular switch, which allowed for the highly specific detection of heavy metal mercury ions (Hg^2+^). When in the presence of Hg^2+^ ions, the signals from Raman probes attach to single-stranded DNA, which will be dramatically enhanced due to the specific structural change of DNA strands—resulting from the interaction between Hg^2+^ ions and DNA bases. This SERS sensor could achieve an ultralow limit of detection (1.35 × 10^−15^ M) for Hg^2+^ detection. In addition, we applied this SERS sensor to detect Hg^2+^ in real blood samples. The results suggested that this SERS platform could be a promising alternative tool for Hg^2+^ detection in clinical, environmental, and food inspection.

## 1. Introduction

Great attention has been paid to heavy metal pollution in our living environment, especially from mercury ions, which can accumulate in the human body [1,2], thus endangering human health. In general, heavy metal can accumulate through food and water, and is easily enriched via the skin and digestive tract, which can lead to damages to the nervous system, cardiovascular system, and the liver [3,4]. The clinical manifestations of chronic mercury poisoning were mainly neurological symptoms, such as headaches, dizziness, and ataxia [5,6]. Therefore, it is of great significance to develop a highly specific and ultrasensitive sensor for detecting the content of Hg^2+^ ions in blood, water, and food samples. At present, there are many traditional methods used to analyze Hg^2+^ ions in samples, such as cold-vapor atomic absorption spectrometry (CV-AAS) [7], the dithizone colorimetric method [8], atomic emission spectrometry [9], and inductively coupled plasma mass spectrometry (ICP-MS) [10]. However, these methods have some deficiencies, such as expensive instruments, complex sample preprocessing, high cost, and vulnerability to the interference of other metal ions. Therefore, it is necessary to develop a more simple, convenient, and sensitive method for the detection of Hg^2+^ ions.

Surface enhanced Raman spectroscopy (SERS) technology is a very powerful trace detection tool that not only has most of the advantages of Raman spectroscopy, which can provide abundant structural information of chemical molecules, real-time and in-situ detection, but also has high sensitivity, simple sample treatment, and high accuracy [11,12]. Fleischmann and Van Duyne et al. found that the Raman signals of the pyrimidine molecules adsorbed on the rough surface of the silver electrode was greatly enhanced [13,14]. With the use of SERS, the Raman signal can be dramatically enhanced by up to 10^14^ times, as compared to normal Raman spectroscopy [15], because of its electronic resonance between optical fields and surface plasmons [16]. Many noble metals, for example, Au, Ag, or Cu can engender strong electromagnetic enhancements [17]. Subsequently, on this basis, a variety of enhanced substrates were developed, such as nanoparticles of different shapes that included nanospheres [18], nanorods [19], nanowires [20], and so on. SERS could overcome the shortcoming of Raman spectra’s low sensitivity and achieve rich structural information that is hard to obtain by conventional Raman spectroscopy. What is more, it can effectively analyze and represent the adsorption orientation, the variation of adsorption state, and the interface information of the compound at the interface [21,22]. With the rapid development of laser technology, nanotechnology, and computer technology, SERS has been widely applied in the material analysis, biomedicine [23], food safety, and in environmental monitoring [11,24]. It should be noted that although mercury, showing a high toxicity to the human body, has been definitively prohibited, it was still widely distributed in water, soil, ambient air, and even food and cosmetics [25,26]. Through breathing, the skin, and the digestive system, it is accumulated and enriched in the organs, especially in the lung, liver, and brain [27,28]. In recent years, the trace detection of heavy metal ions has attracted more and more attention. Several groups have proposed various methods for detecting Hg^2+^, which is based on different SERS substrates. Liu’s group has developed a one-step, room temperature, and colorimetric method by using oligonucleotide-tethered AuNPs probes and a linker oligonucleotide with a number of T-T mismatches, resulting in the formation of particle aggregates accompanied colorimetric responses with the addition of Hg^2+^ into the solution [29]. Yang et al. have provided an approach for the visual and fluorescent sensing of Hg^2+^ in aqueous solution, which is based on the Hg^2+^-induced conformational change of a thymine (T)-rich single-stranded DNA (ssDNA), and the difference in electrostatic affinity between ssDNA and double stranded (dsDNA) with gold nanoparticles [30]. His group used the resultant Ag–Au NPs@Si as a substrate for detecting Hg^2+^ ions with a low detection limit of 100 fM, and had a good linear relationship [31]. Herein, we developed a new method with more power to detect Hg^2+^ ions with a low detection limit of 1.35 × 10^−15^ M in human blood samples.

Since mercury ions (Hg^2+^) can specifically form a strong and stable thymine-Hg^2+^-thymine complex (T-Hg^2+^-T) with thymine bases (T) [29,30], the oligonucleotide probes can be designed based on this principle to detect mercury ions. For the first time, the functionalized silver film was used as a SERS substrate for Hg^2+^ ions detection. Herein, a facile SERS substrate was prepared for Hg^2+^ detection by silver mirror reaction (SMR), which was one of the most effective technologies for preparing micro-nanometallic materials, and has the features of cost-effectiveness, high uniformity, simplicity of operator, and so on [32,33]. By taking the advantages of the specific binding of oligonucleotides and silver-based Raman-active substrate, in this work, we proposed a simple and highly specific method to detect the content of Hg^2+^ ions in complex biomatrices, and aim to overcome the drawbacks of traditional detection methods of Hg^2+^ ions, such as poor specificity and sensitivity.

## 2. Materials and Methods

### 2.1. Materials and Reagents

Silver nitrate (AgNO_3_), ammonium hydroxide (NH_3_·H_2_O), glucose (C_6_H_12_O_6_), absolute ethyl alcohol (C_2_H_6_O), Rhodamine 6G (R6G), glass slides (0.5 cm × 0.5 cm), sodium chloride (NaCl), Phosphate-buffered solution (PBS, PH = 7), and DNA probe (Cy5-TTCTTTCTTGGGGTTGTTTGTT-SH) were purchased from the Sangon Biotech (Shanghai, China). CaCl_2_, CuCl_2_, MnCl_2_, AlCl_3_, ZnCl_2_, NiCl_2_, CdCl_2_, FeCl_3_, MgSO_4_, and HgCl_2_, were analytical reagents and had not been further purified. The standard solutions of Hg^2+^ ions were diluted by deionized water. The blood samples were obtained from the Fujian Province Tumor Hospital, Fuzhou, China. All of the chemical regents were analytical grade, and all solutions were prepared with ultrapure water.

### 2.2. Instrumentation

The SERS spectra was obtained using a confocal Raman micro-spectrometer (Renishaw, London, UK) under the excitation of a 785 nm laser in the range of 400–1800 cm^−1^, which was performed by using the software package WIRE 2.0 (Renishaw, London, UK). The laser power that we chose was about 5 mW, employing typically 10 s exposure time, and a 2 cm^−1^ resolution of the Raman spectra. We used a microscope with a Leica ×20 objective to obtain the spectral signal in posterior scattering geometry. The wavelength was calibrated by using the 520 cm^−1^ vibration band of the silicon wafer. Scanning electron microscopy (SEM) images were taken with a JSM-6380LV scanning electron microscope (JEOL, Tokyo, Japan).

### 2.3. Preparation of Samples

#### 2.3.1. The Preparation of Silver Ammonium Solution

The concrete steps were as follows. The ammonia solution was added in the 3 mL 2 wt% silver nitrate solution, drop by drop, to prepare the silver ammonia solution. A brown precipitate was first produced, and ammonia was continuously added dropwise until the precipitate completely dissolved [34]. Then, a drop of 6 wt% glucose solution was added and shaken.

#### 2.3.2. The Preparation of Standard Solutions of DNA Probe (Cy5-α-SH) and Rhodamine 6G (R6G)

Firstly, the PBS buffer solution was used as a solvent to dissolve the DNA probe (Cy5-α-SH) and prepared the solution with a concentration of 100 μM. Then, the initial concentration was diluted to the concentration of 1 μM and was used in this experiment.

In addition, weighing R6G powder was dissolved in deionized water and was transferred to a volumetric flask, setting to a constant volume and shaking. The concentration of the solution was 10^−4^ M and was stored for later use.

#### 2.3.3. The Formulation of Standard Solutions of Hg^2+^

Briefly, Hg^2+^ solution was prepared with ultrapure water at an initial concentration of 10^−3^ M. The solution was then shaken completely to form a highly dispersed solution. Following this, the solution was diluted to a concentration from 10^−6^ to 10^−15^ M.

#### 2.3.4. Preparation of Human Plasma Samples

After 12 h of overnight fasting, a single 3 mL peripheral blood sample was obtained from the study subjects between 7:00 a.m.–8:00 a.m. with the use of coagulant. Blood cells were removed by centrifugation at 2000 rpm for 15 min to obtain the blood plasma.

### 2.4. Preparation of SERS Substrate

#### 2.4.1. Cutting and Cleaning of the Slides

First of all, sheet glass was cut into small pieces of 0.5 cm in length and width (0.5 × 0.5 cm). The prepared small pieces of glass were degreased, soaked with chromic acid for more than 2 h, and then washed with ultrapure water, followed by an ultrasound for 30 min in anhydrous ethanol and washed with ultrapure water 5–6 times.

#### 2.4.2. Synthesis of the Silver Film

The treated small glass flakes were put into the prepared silver ammonium solution. The reaction time was 15 min and the temperature was 70 °C, which was the most significant aspect of accurately controlling the reduction reaction time and temperature. As a result, a layer of silver was deposited on the slide. It was taken out of the glass, washed 4–5 times with ultrapure water, and dried in a slow nitrogen flow to prepare for the next modification with the DNA probes.

### 2.5. Reproducibility Detection of SERS Substrate

Firstly, R6G was used as the target molecules, and the prepared standard solution, was randomly dripped on the substrate of Ag-film. After drying, it was placed under a microscope to collect the Raman spectrum by a ×20 objective.

In addition, the reproducibility of the substrate of Ag-film was verified by using the DNA probe as the target molecules, and the SERS substrates of the assembled probe chain, were immersed in the standard solution of Hg^2+^ with a concentration of 10^−8^ M. After drying in a gentle nitrogen flow, a ×20 objective was used to collect the corresponding SERS spectra.

### 2.6. Construction of Probes for Detection of Mercury Ions (Hg^2+^)

The prepared substrate constructed the SERS sensor that specifically responded to Hg^2+^ by conjugating with single-stranded DNA (Cy5-α-SH). The assembly process was as follows. The prepared SERS substrates were immersed in the assembly buffer (PH = 7.0) for 12 h, which contained 1 μM DNA probes (Cy5-α-SH) with a double labeling of dye molecules Cy5 and thiol molecules, and 10 mM phosphoric acid solution. Then, the substrates were placed in phosphate buffered saline solution, including 0.1 M NaCl, overnight to ensure that sufficient stem-loop DNA self-assembled with the silver film via Ag-S bonds. Next, the substrates were again washed five times with phosphate buffered solution (PBS) and dried with a gentle nitrogen flow. 

### 2.7. Detection of Mercury Ions (Hg^2+^)

The assembled substrates were immersed in the standard solution with different concentrations of Hg^2+^ for 120 min, and taken out and washed 3 times. After drying, a SERS measure was performed to obtain the corresponding Raman spectra by using a 785 nm laser with a power of 5 mW, and the control baseplates were also observed and detected by a Leica ×20 objective.

### 2.8. Detection of Hg^2+^ Ions in Blood Samples

The blood samples were collected from the Fujian Province Tumor Hospital. Firstly, the blood was taken from healthy volunteers, and the blood plasma was obtained by centrifugation. The main components of plasma include 90–92% of water, and the other 10% are mainly solute plasma proteins. The obtained plasma interacted with the assembled substrate for 2 h, taken out and washed 3 times, and then dried under nitrogen to perform SERS detection. Next, we added the standard solution of Hg^2+^, with the concentration of 50 nM and 100 nM, to the plasma samples by the standard addition method. Finally, the assembled substrates were immersed in the prepared plasma samples, and then a SERS measurement was performed. Each sample was measured 3 times to obtain an average.

## 3. Results and Discussion

**Figure 1:**
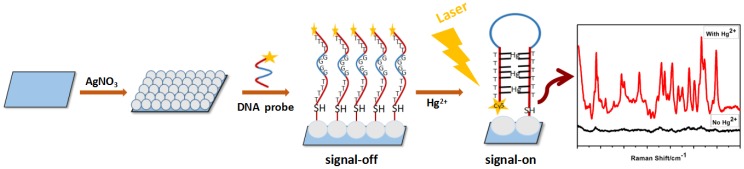
Schematic illustration of the mechanism of the surface-enhanced Raman scattering (SERS) sensor for the detection of mercury ions (Hg^2+^), based on the T-Hg^2+^-T coordination.

The proposed SERS sensor was based on the specific binding feature of the T-Hg^2+^-T coordination, and the Cy5-labeled DNA strands (Cy5-α-SH) can be self-assembled on the silver film substrate via Ag-S bonds [6,29]. In this study, DNA strands were used as a switch for the Raman signal, which indirectly indicated whether there were mercury ions (Hg^2+^) in the analyte, as shown in Figure 1. The SERS substrate of the Hg^2+^ sensor was the Ag film with a uniform surface via silver mirror reaction. Next, the probe chains were modified on the substrate. Briefly, the substrates were immersed in the solution of the probe chains, then removed and dried. Here, the principle of the probe molecule chains, as a signal switch to detect Hg^2+^ ions, was that, in the absence of Hg^2+^ ions, the DNA probes showed an “open” conformation (signal-off), and Cy5-tagged enzyme strands were far away from the substrate, thus producing a weak Raman signal. However, in the presence of the Hg^2+^ ions, the former “open” structural conformation would change into a “hairpin” structure, which was formed by Hg^2+^ ions bonding with thymine bases (T) [30,35]. This conformational change resulted in a shorter distance between Cy5 labeled molecules and the surface of the Ag-film, leading to a strong Raman signal (signal-on). We also investigated the effect of the concentration of single-stranded DAN and the space occupied by the probes of the stem-loop structure on the experiment. He’s group [36,37] employed gold nanoparticles (AuNPs), decorated with a silicon nanowire array (SiNWAr), for surface-enhanced Raman scattering (SERS) substrates to detect Hg^2+^. By comparing the relationship between fluorescence intensity and the corresponding concentration of Cy5-ssDNA, we can observe that, with the increase of Cy5 modified single-stranded DNA from 0 to 1 μM, fluorescence intensity gradually enhances and tends to be a saturated value. Similarly, the size range of the synthesized Ag-film substrate in our experiment was at the micro-nanometer level and obviously exhibited a three-dimensional spatial configuration, which was formed by silver clusters in the shape of a polyhedron at the top. In addition, the size of the substrate they used was the same as ours (0.5 × 0.5 cm), and therefore, the concentration of ss-DNA was 1 μM in our experiment when assembling DNA molecules on an Ag-film substrate. We predicted that there was enough space for the ss-DNA to form a “hairpin” structure on a SERS substrate. In the experiment, the sensitivity of the analysis system would be affected by the length of the DNA strands and the number of T-T mismatched bases. Yang’s group proposed a method for visual and fluorescence sensing of Hg^2+^ in aqueous solution, and also made a corresponding study of the length of single-stranded DNA and the number of T-T mismatched bases with the interaction of Hg^2+^ ions [30]. By comparing the fluorescence response of a different number of the T-T mismatch sites to the different concentrations of Hg^2+^, the DNA chains of the 7 mer T-T mismatch sites had the most significant responses to the fluorescence of Hg^2+^. In addition, comparing the fluorescence response of single-stranded DNA with different chain lengths to different concentrations of Hg^2+^, it was found that the corresponding fluorescence of Hg^2+^ was decreased with the increase of chain length. Importantly, the greatest advantage of the DNA strand of the 7 mer T-T mismatch sites was that they were more sensitive to the low concentration of Hg^2+^ ions—so, the DNA strand currently used is optimal.

**Figure 2:**
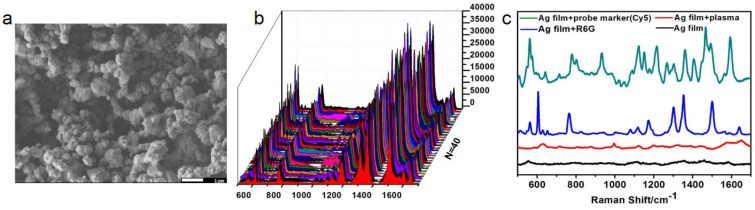
(**a**) The SEM image demonstrating the morphology of the Ag-film substrate. (**b**) SERS spectra of R6G with a concentration of 10^−4^ M were collected from 40 random points on the resultant substrates. (**c**) The spectral signal of plasma detected on a silver-film substrate (red), SERS spectra of R6G (10^−4^ M) distributed on silver film substrate (blue), spectrum of the Cy5 reporter (green), and background signal of the synthesized Ag film substrate (black).

In our experiment, the Ag film was synthesized by silver mirror reaction and was used as a SERS substrate. The Ag film formed was analogous to a 3-D spatial structure, which was evidently demonstrated in the scanning electronic microscopy (SEM) image (Figure 2a). Close examination revealed that there were many nano-cavities and nano-gaps formed by the stacking of closely adjacent nanoparticles. Many papers have shown an extremely strong local field enhancement in the gap between two closely spaced silver nanoparticles [38,39]. Therefore, the 3-D Ag-film substrates may generate a lot of effective hot spots and a strong SERS response for DNA detection. In addition to this, our method is characterized by the features of cost-effectiveness and facile synthesis, as well as being easy to repeat. The spatial morphology is evidently demonstrated in the scanning electronic microscopy (SEM) image (Figure 2a).

The SERS technology has the advantages of high accuracy, easy operation, and simple sample pretreatment [11,12]. Therefore, SERS detection plays an active role in the rapid detection of trace level and heavy metal ions. However, the reproducibility of SERS detection is a common problem that has plagued researchers. In order to evaluate and verify the Ag film-based substrate used in our experiments, we used R6G as target molecules on the synthesized substrates, and SERS measurements were performed from 40 random spots. The resulting SERS spectra are shown in Figure 2b, which clearly presents a relatively uniform Raman spectrum, and the Relative standard deviation (RSD) value of the Raman spectrum of R6G was 15.1%—by selecting a Raman peak of R6G at 1362 cm^−1^. Typically, the SERS spectra of R6G, with a concentration of 10^−4^ M, was measured on a prepared silver film substrate (the blue line), as shown in Figure 2c, the red line is the spectral signal of plasma detected on an Ag-film substrate showing that the plasma has a lesser background interference signal on the silver film, and the green line was the spectrum of SERS reporter Cy5. The background signal of this substrate is the black line, showing that the synthesized Ag film had little background interference, enabling a reliable SERS detection using this substrate.

**Figure 3:**
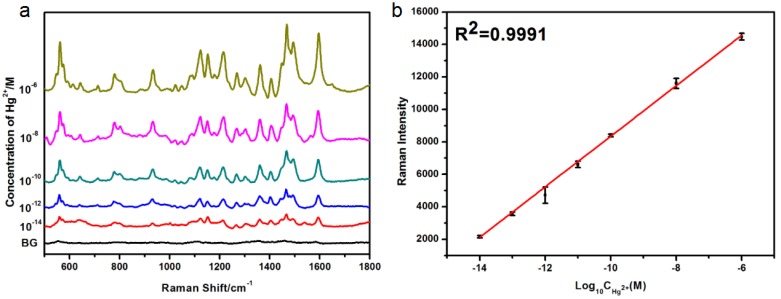
(**a**) SERS spectra of Cy5 with different concentrations of Hg^2+^ in distilled water ranging from 1.0 × 10^−14^ to 1.0 × 10^−6^ M (BG = blank control), (**b**) standard curve of Raman intensity of Cy5 at 1595 cm^−1^ with logarithmic Hg^2+^ concentrations from 10^−14^ to 10^−6^ M.

By utilizing this Cy5 labeled, single-stranded DNA, with SERS sensor as a signal switch, we measured different concentrations of Hg^2+^ ions in the standard solution; a series of SERS spectra of Hg^2+^ ions with concentrations from 1.0 × 10^−14^ to 1.0 × 10^−6^ M could be obtained. Importantly, we were able to clearly observe the characteristic peaks of Cy5 reporters (Figure 3a). According to the principle of T-Hg^2+^-T coordination [29,35], when there was no mercury ions, the signal switch was in the “closed state”, so the SERS signal of the Cy5 reporters was very weak—as shown by the black line in Figure 3a. However, when the Hg^2+^ ions were presented, in the measured solution, the signal switch was “turned on” and the SERS signal of the Cy5 was immensely enhanced. Furthermore, with the concentration of the Hg^2+^ ions increasing gradually, the SERS intensity of Cy5 was increased simultaneously, which can thus be used as an indicator for the concentration of the Hg^2+^ ions. In order to accurately demonstrate the effect of concentrations of Hg^2+^ ions on the SERS intensity, we monitored the intensity of the SERS peak at 1595 cm^−1^ (assigned to C=N stretching modes). As shown in Figure 3b, the SERS intensity of Cy5 at 1595 cm^−1^ was increased from 2153.6 to 14,478.8 (a.u.), and the concentration of Hg^2+^ gradually increased from 10 fM to 1 μM. The standard curve was achieved as *y* = 1575.7*x* + 24,019, it should be noted that the calculated correlation coefficient was *R*^2^ = 0.9991, indicating that it exhibited a good linear relationship between the logarithm of concentration of Hg^2+^ and SERS intensity of the probe, and the limit of detection (LOD) for Hg^2+^ was 1.35 × 10^−15^ M. We adopted a method of signal-to-noise ratio (S/N) of approximately 3:1, which was generally considered to be acceptable for estimating the limit of detection (LOD). Taking the standard solution of Hg^2+^ with a concentration of 10^−14^ M as an example, the SERS intensity of the 1595 cm^−1^ peak, and the background baseline, were selected to calculate, and the LOD was 1.35 × 10^−15^ M. Compared with other reports based on the mechanism of T-Hg^2+^-T coordination [30,40], a much lower LOD for mercury ions using SERS measurement can be obtained using this novel sensor with single-stranded DNA as a signal switch. The LOD obtained was seven orders of magnitude lower, as compared to the defined limit (10 nM) in drinkable water by the United States Environmental Protection Agency (USEPA). At a higher magnification of the SEM image, as shown in Figure 2a, we can clearly observe that the size range of the synthesized silver film substrate was at the micro-nanometer level and obviously exhibited a three-dimensional spatial configuration that was formed by silver nanoparticles and silver clusters in the shape of a polyhedron at the top [41,42]. It is well known that a powerful and effective SERS substrate was attributed to the huge amount of micro-/nanoscale structures of polyhedral and nanoscale junctions [38,39], which thereby formed a great deal of SERS “hot spots” that enhanced the intensity of the electromagnetic field, resulting in the tremendous enhancement of the SERS signal. It was precisely for this reason that the limit of detection (LOD) for Hg^2+^ ions in our experiments can be as low as 1.35 × 10^−15^ M. In addition, we also made a standard curve of the SERS intensity of Cy5 at 1362 cm^−1^ (attributed to the methine chain deformation of Cy5) with logarithmic Hg^2+^ concentrations from 10^−14^ to 10^−6^ M, and the correlation coefficient was *R*^2^ = 0.9990. By comparing the correlation coefficients of the two peak positions, we can draw a conclusion that the values of *R*^2^ were almost the same, and it also proved that the correlation between the SERS intensity and concentrations of Hg^2+^ ions is good.

**Figure 4:**
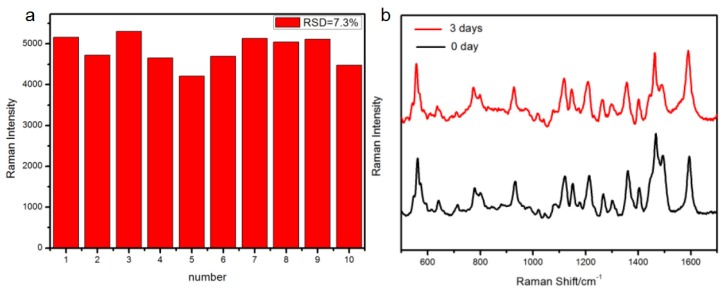
(**a**) Relative standard deviation (RSD) of specific Raman modes at 1362 cm^−1^ of the 10 random points; and, (**b**) SERS signals of the labeled molecule (Cy5) on the functionalized Ag-film substrates under the exposure to the common air environment for 0 days and 3 days.

To evaluate the reproducibility of the Hg^2+^ sensor, after modifying the DNA probes on the Ag-film substrates, we used the standard solution of Hg^2+^ with a concentration of 10^−8^ M to immerse the substrate, and then measured the SERS spectrum at ten randomly selected spots. The RSD value was 7.5%, from a comparison of the intensity of the Raman peaks at 1362 cm^−1^ in Figure 4a, which bring reliable SERS detection. In addition, in order to verify the stability of the sensor, we placed the substrate in the air for three days where it interacted with a standard solution of Hg^2+^ at a concentration of 10^−8^ M. Figure 4b shows the SERS signals of the labeled molecule from substrates, exposed to the common air environment for zero days and three days, and we can clearly see that the Raman signals did not significantly attenuate. Consequently, we can conclude that the SERS sensor exposed to air over a short time could not lead to an obvious attenuation of SERS signals. This can be explained as follows. Under mild oxidizing conditions, the formation of a silver oxide layer after 20 h of exposure time was not detected by X-ray photoelectron spectroscopy (XPS). Then, by using UV to detect again, the relevant data showed no significant shift in the surface plasmon bands of immobilized Ag NPs after three days of exposure to environmental air [43,44]. Sukhishvili’s group followed the idea that molecular oxygen does not oxidize silver under ambient conditions, and the ozone is considered to be the main oxidant for starting the oxidation process of silver. In addition, studies on the change of plasma absorption bands show that chemical enhancement was the main reason for the attenuation of SERS signals by exposure to ambient air. However, in our experiment, since there was no direct contact between the labeling molecule and the SERS substrate after the formation of the “hairpin” structure [31,45], the enhancement effect of the SERS signals mainly depend on the electromagnetic enhancement, and not the chemical enhancement. Therefore, in the mild environment of the laboratory, silver oxidation has little effect on the detection results within a short time.

**Figure 5:**
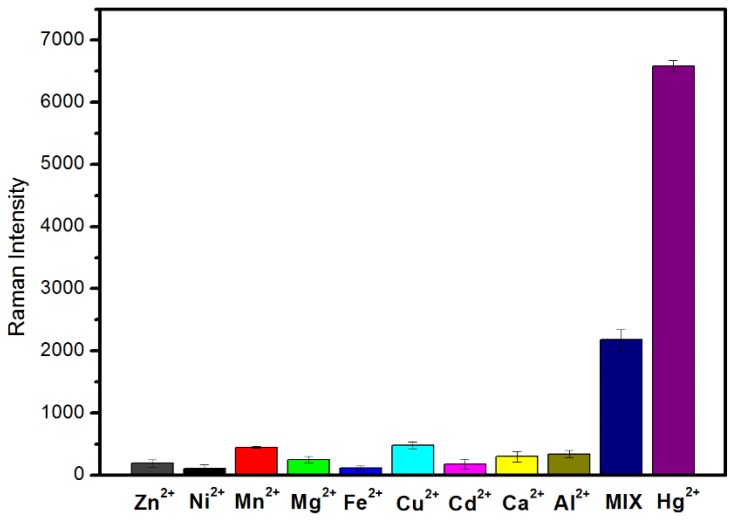
Specificity of the SERS sensor for Hg^2+^ detection. The concentration of Hg^2+^ was 10 nM and other interference metal ions were 1 μM. Additionally, all metal ions were mixed together, including the Hg^2+^ ions (mix).

To verify the high selectivity and specificity of this SERS sensor, we also measured other metal ions using this sensor. By comparing the SERS intensity of the 1362 cm^−1^ peaks for the Cy5-labeled molecules, as shown in Figure 5, the strong Raman signal can only be observed in the presence of Hg^2+^ ions, rather than any other metal ions. The mixture of ions showed a smaller Raman intensity than the Hg^2+^ only. This is explainable. In the experiment, when Hg^2+^ ions were detected separately, the concentration was 10 nM and the volume was 200 μL, and the amount of Hg^2+^ ions substance (n) was 2 × 10^−10^ mol. However, the concentration of Hg^2+^ used in the preparation of the mixed solution was 10 nM and the volume was 60 μL, the n of Hg^2+^ was 6 × 10^−11^ mol. The *n* of Hg^2+^ ions in the mixed solution was only one third of the original. The less amount of Hg^2+^ ions, the less Raman-labeled molecules can be detected, thus the intensity of the Raman spectrum will be reduced. It should be noticed that the spectral intensity of Hg^2+^ was still distinctly stronger than in other metal ions, even though the concentration of other interference metal ions was 100 times higher than the Hg^2+^ ions. This demonstrated that this sensor was highly selective for Hg^2+^ ions because Hg^2+^, and probe chains, had considerable binding affinity to form the stable T-Hg^2+^-T complexes.

To evaluate the practicability of this method, we used it to detect the Hg^2+^ ions in real human blood samples. The method we used was the standard addition method. We measured the content of Hg^2+^ ions in the blood of a healthy person, and the prepared blood samples added the standard solutions of Hg^2+^ ions with different concentrations. The SERS measurement for each specimen was repeated three times. The related statistics are shown in Table 1. The resulting recovery rate for Hg^2+^ ions was 91–104%, showing a remarkable recovery rate. The results showed that this method has good prospects and applicability for the detection of Hg^2+^ in body fluid.

## 4. Conclusions

In summary, an ultra-sensitive, highly specific SERS sensor was developed for detecting Hg^2+^ ions in solution, and in complex human body fluids, using single-stranded DNA as the signal switch. In the presence of Hg^2+^ ions, there was a transformation from an “open” single-strand of DNA, to a “hairpin” configuration via the T-Hg^2+^-T coordination. Additionally, using the silver mirror reaction, we prepared a highly efficient SERS substrate, which was characterized with a homogeneous 3D micro-nano structure, thus achieving enormous “hotspots”. Results show that this sensor has both high sensitivity (a limit of detection of 1.35 × 10^−15^ M) and specificity for Hg^2+^ detection. Even for real blood samples, this sensor still presented a high performance for Hg^2+^ detection, demonstrating the great potential of this senor for heavy metal ions detection in real human biofluids.

## Figures and Tables

**Table 1:** Determination results of Hg^2+^ in human blood samples.

Sample	Spiked Concentration (nM)	Detected Concentration (Mean ± SD, nM, *n* = 3)	Recovery (%)
Original blood	0	(2.0 × 10^−4^) ± 1.67	
blood sample 1	50	48.89 ± 1.37	91–104
blood sample 2	100	97.66 ± 6.6	95–101
